# Beta-Blockers and Berberine: A Possible Dual Approach to Contrast Neuroblastoma Growth and Progression

**DOI:** 10.1155/2020/7534693

**Published:** 2020-08-12

**Authors:** Maura Calvani, Angela Subbiani, Gennaro Bruno, Claudio Favre

**Affiliations:** ^1^Department of Paediatric Haematology-Oncology, A. Meyer University Children's Hospital, Florence, Italy; ^2^Department of Health Sciences, University of Florence, Florence, Italy

## Abstract

The use of nutraceuticals during cancer treatment is a long-lasting debate. Berberine (BBR) is an isoquinoline quaternary alkaloid extracted from a variety of medicinal plants. BBR has been shown to have therapeutic effects in different pathologies, particularly in cancer, where it affects pathways involved in tumor progression. In neuroblastoma, the most common extracranial childhood solid tumor, BBR, reduces tumor growth by regulating both stemness and differentiation features and by inducing apoptosis. At the same time, the inhibition of *β*-adrenergic signaling leads to a reduction in growth and increase of differentiation of neuroblastoma. In this review, we summarize the possible beneficial effects of BBR in counteracting tumor growth and progression in various types of cancer and, in particular, in neuroblastoma. However, BBR administration, besides its numerous beneficial effects, presents a few side effects due to inhibition of MAO A enzyme in neuroblastoma cells. Therefore, herein, we proposed a novel therapeutic strategy to overcome side effects of BBR administration consisting of concomitant administration of BBR together with *β*-blockers in neuroblastoma.

## 1. Neuroblastoma

Neuroblastoma (NB) is the most common extracranial childhood solid tumor, which arises from the embryonic primary neural crest cells during development. NB accounts for approximately 8% of all diagnosed pediatric cancers and causes 9-15% of all cancer-related deaths in childhood. NB is clinically heterogeneous, with prognosis spanning from favorable outcome in low and intermediated risk to poor outcome in high-risk cases [1]. In addition, biological and chromosomal markers are associated with NB aggressiveness, including the amplification of the proto-oncogene *MYCN*, dysregulation of anaplastic lymphoma kinase (ALK), and genetic aberration [2, 3]. *MYCN* amplification occurs in approximately 25% overall of NB cases and particularly in 40% of the high-risk cases, representing the major prognostic marker associated with unfavorable clinical outcomes [4, 5]. Several studies have reported that *MYCN* overexpression in peripheral neural crest is sufficient to initiate NB in a mouse model [6], while *MYCN* downregulation increases differentiation and apoptosis, and represses proliferation and tumor growth *in vivo* [7–9]. NBs with low MYCN levels have better responses to chemotherapy and overall survival. MYCN amplified NB tumors that were originally responsive to chemotherapy [10], tend to have an early relapse, and then become resistant [11]. ALK mutation occurs in approximately 14% of high-risk cases and is associated with a relapse of NB [12, 13]. *In vivo* studies have demonstrated the interaction between ALK mutation and *MYCN* amplification contributing to NB progression [14, 15]. Indeed, ALK induces the transcription of *MYCN* [16], or sporadically, when coamplified with *MYCN*, ALK leads to an increased expression of ALK protein, resulting in a poor prognosis of NB patients with both *MYCN* amplification and ALK mutation or amplification [17]. According to the INRG staging system, NB tumors are classified in different risk groups: L1 for localized tumors that do not involve vital structures as defined by the list of image-defined risk factors (IDRFs); L2 for locoregional tumors with one or more IDRFs; M for distant metastatic disease; M2 for metastatic disease in children younger than 18 months with metastasis confined to skin, liver, and/or bone marrow [18]. Instead, a combination of factors, including age at diagnosis, stage, tumor histology, tumor cell ploidy, and MYCN status, has been used to stratify patients into three pretreatment groups, low-, intermediate, and high-risk, according to The Children's Oncology Group (COG). Surgery is the main treatment for low-risk NB with or without postsurgery chemotherapy, with a 5-year survival rate higher than 95%. Treatment for high-risk tumors usually includes a combination of chemotherapy, radiotherapy, bone marrow or stem cell transplant, surgery, immunotherapy, and administration of oral retinoid. However, for high-risk NB, the 5-year survival rate is still approximately 40% to 50%.

## 2. Beta-Adrenergic Receptors

Beta-adrenergic receptors (*β*-ARs) belong to the superfamily of G-protein-coupled receptors (GPCRs) and are divided into three receptor subtypes, *β*1-, *β*2-, and *β*3-AR. All three *β*-ARs are known to regulate many physiopathological processes in humans and animals, following their activation elicited by the catecholamines, such as noradrenaline or adrenaline [19, 20]. Examples of typical responses mediated by *β*-ARs are vasodilation, cardiac functions, and thermogenesis. Different *β*-ARs are expressed in numerous cell types and tissues, where they are able to activate downstream pathways that can be distinct for each subtype of *β*-ARs or even overlapped. However, while the *β*1-AR and *β*2-AR are almost ubiquitous in tissues, the *β*3-AR subtype has a more restricted pattern expression in humans [21], together with a unique pharmacology compared to *β*1-AR and *β*2-AR [22].

Due to the broad expression of *β*-ARs in different organs, and their ability to regulate several biological processes, the use of both agonists and antagonists of *β*-ARs has become a widespread strategy in pharmacology. Indeed, up to date, the FDA has approved 19 *β*-blockers as therapeutic agents to different diseases: *β*2-AR agonist salbutamol and formoterol for asthma and chronic obstructive airway disease, *β*1-AR antagonist metoprolol and bisoprolol for coronary heart disease and arrhythmias, *β*1-AR agonists such as dobutamine for acute heart failure, *β*1-/*β*2-AR antagonist carvedilol for chronic heart failure, *β*3-AR agonist mirabegron for overactive bladder syndrome, and many others [23]. High concentrations of catecholamines and their metabolites found in patients affected by NB, may suggest that studying of the adrenergic system, and in particular of the *β*-ARs, could represent a promising option for alternative treatments against this cancer.

## 3. Beta-Adrenergic Receptors in Cancer

In 1989, Schuller and Cole reported that the nonselective *β*-ARs agonist isoprenaline was able to induce proliferation of lung adenocarcinoma cells; on the contrary, the nonselective *β*-ARs antagonist propranolol counteracted the effect of *β*-ARs stimulation [24]. This was the first evidence clearly showing the role of *β*-ARs in tumor growth, which was further confirmed by numerous studies on different tumor types. To date, the relationship between stress and tumor progression has been clearly demonstrated through numerous preclinical and clinical evidences [25]. In particular, catecholamine release by the sympathetic nervous system is able to activate *β*-ARs, which sustain numerous tumor-related signaling pathways involved in tumor progression, metastasis, and response to treatment, including inflammation, angiogenesis, resistance to apoptosis, tumor cell invasion, and epithelial to mesenchymal transition [26]. Indeed, the three *β*-ARs are expressed and involved in the pathogenesis and/or progression of different tumors, from benign form such as the infantile hemangioma [27, 28] to several malignant types including breast cancer [29], ovarian cancer [30, 31], melanoma [32, 33], colon cancer [34], and many others. Accordingly, previous studies showed that pharmacological *β*-ARs blockade was efficient to suppress stress-induced enhancement of tumor progression in melanoma [35] as well as in breast [36], prostate [37] cancers, and leukemia [38]. In several murine experimental models, in different types of tumors, reduced tumor growth and progression following propranolol administration suggested a role for *β*1- and/or *β*2-ARs as key mediators of stress-induced tumorigenic events. Specifically, propranolol was able to affect tumor growth in numerous malignancies such as in ovarian, prostate, pancreatic, and other cancers [39–41]. In many of these studies, the effects observed following propranolol administration relied on the *β*2-AR blockade rather than on *β*1-AR involvement. Therefore, until few years ago, the *β*2-AR was recognized as the main *β*-AR subtype involved in the regulation of tumor-related pathways [42]; however, recent studies have shown the crucial role of the *β*3-AR subtype in cancer biology. In particular, *β*3-AR gene polymorphisms have been associated with decreased risk or susceptibility to some cancers including breast, endometrial, and gallbladder cancer [43–45]. In a case series study, an aberrant *β*3-AR mRNA upregulation was found related to the neoplastic transformation of colorectal cancer [34]. Through a xenograft murine model of prostate cancer, Magnon et al. demonstrated that genetic deletion of stromal *β*2/3-ARs prevented the early phases of tumor development and tumor cell dissemination [46]. Moreover, numerous preclinical studies on melanoma have shown that *β*3-AR is able to sustain protumoral activities in tumor cells but also in stromal cells of the tumor microenvironment, and its blockade exerts a crucial antitumor action by affecting multiple signaling pathways [33, 47, 48]. These experimental evidences confirmed the critical involvement of *β*-ARs in regulating tumor progression in many malignancies and suggested that *β*-ARs and their related pathways must be further investigated in order to explore new therapeutic interventions in many tumor diseases.

## 4. Beta-Adrenergic Receptors in NB

Patients diagnosed with NB usually have elevated hematic and urinary concentration of catecholamines or their metabolites. Both urinary homovanillic and vanillylmandelic acid (HVA and VMA) are catecholamine metabolites that have been used as biomarkers in the diagnosis and follow-up of patients with NB [49, 50]. Elevated levels of these metabolites suggest, therefore, that the adrenergic system could play an important role in regulating NB biology; indeed, recent findings have demonstrated that *β*-ARs modulation, thorough the use of *β*-blockers, affects NB tumor growth and progression. The effects of the nonselective *β*-blocker propranolol administration have been studied through *in vitro* and in *vivo* experimental models [51]. In particular, in a panel of fifteen human neuroblastoma cell lines, propranolol was able to inhibit NB tumor growth, survival, and proliferation and to induce apoptosis via activation of p53 and p73 signaling. These effects were mediated by the *β*2-AR, but not *β*1-AR, and accordingly, all analyzed cell lines expressed the *β*2-AR protein. In the same study, these results were confirmed thorough an *in vivo* xenograft model (NOD/SCID mice inoculated with human SK-N-AS cells), in which administration of propranolol (1 mg/kg) resulted in a decreased tumor growth compared to a control group. Furthermore, propranolol was also synergistic with the topoisomerase I inhibitor SN-38 in reducing tumor growth of NB *in vivo* [51]. Accordingly, a second study [51] showed that the three *β*-blockers carvedilol (a mixed *α*/*β*-blocker), nebivolol (a selective *β*1-blocker), and propranolol (nonselective *β*-blocker) exhibited potent anticancer properties on NB. Interestingly, *in vitro*, the three *β*-blockers potentiated the antiangiogenic, antimitochondrial, antimitotic, and, ultimately, proapoptotic effects of vincristine in humans (BE (2) C, SHEP, SK-N-SH) and mouse-derived (NH02A) NB cell lines. In TH-MYCN transgenic mice, the administration of *β*-blockers transiently reduced tumor growth; more importantly, *β*-blockers used in combination with vincristine, were able to increase the antitumor effect of vincristine compared with this chemotherapeutic agent alone. The synergistic effect of combining *β*-blockers and vincristine was associated with an increase in tumor angiogenesis inhibition and ultimately resulted in a four-fold increase in median survival, as compared with vincristine alone [52]. In a recent study, the *β*3-AR subtype, whose expression and function on NB had not yet been investigated to date, was found as crucially involved in NB tumor growth and progression [53]. In particular, the expression of *β*3-AR was evident in human (SK-N-BE, BE (2) C) and murine (Neuro-2A) NB cell lines, as well as in biopsies obtained from patients affected by NB. *β*3-AR blockade *in vitro*, using the antagonist SR59230A, was able to decrease cell proliferation and increase neuronal differentiation at the expense of stemness traits. Moreover, the pharmacological blockade of *β*3-AR strongly affected NB tumor growth *in vivo* in a syngeneic NB model, confirming the data obtained *in vitro* [53]. These results highlighted for the first time the crucial role of the *β*3-AR subtype in regulating tumorigenesis of NB. Taken together, all these preclinical data suggest that *β*-blockers have an important role in counteracting NB tumor growth and progression, and thus they should be considered for the treatment of patients diagnosed with NB either alone or in combination with other therapeutic agents.

## 5. Nutraceutical Compounds and NB

Despite advances in diagnosis and therapy, NB still remains a challenge in terms of recurrence and survival. Standard therapies against NB involve radiotherapy and chemotherapy that are able to induce significant responses or remission in the majority of patients. However, the side effects of these treatments due to damage and toxicity do not prevent recurrence in most patients with high-risk factors [54]. Therefore, the research of novel therapeutic agents to improve treatment outcomes in NB is deeply important. Natural compounds derived from animals, microorganism, and in particular from plants have been used and have demonstrated significant efficacy in the prevention and treatment of various human disease, including cancer. Various studies have demonstrated that the combination of chemotherapeutic agents and resveratrol (RV), a polyphenol found in red wine with antioxidant, anti-inflammatory [55], cardioprotective [56], and anticancer activities [57, 58], lead to the inhibition of tumor growth and enhanced antitumor effect compared to chemotherapy alone [59]. Recently, *in vitro* and *in vivo* studies have reported the effects of RV in NB proliferation and progression. van Ginkel et al. have shown that RV treatment reduced tumor growth in NGP and SK-N-AS xenograft model of NB, without evidence of toxic effects and accumulation of RV in serum, liver, or other tissues. In SK-N-AS, NGP, SH-SY5Y NB cell lines, RV treatment induced cell cycle arrest in G1 and S phase and caused the collapse of the mitochondrial membrane potential with the release of cytochrome *c*, activation of caspase-3 and caspase-9 protein, leading to an enhanced apoptosis and a decreased cell viability and proliferation [60]. In B103 NB cells, RV treatment induced cell cycle arrest in S phase via the downregulation of the cyclin D1, and induced apoptosis through the reduction of the antiapoptotic proteins Bcl-2, Bcl-xL, and Mcl-1, in a dose-dependent manner [61]. Graham et al. have demonstrated that the combined treatment with RV and 2-Deoxy-D-glucose (2-DG) induced the activation of caspase-3 protein and apoptotic cell death, compared to 2-DG treatment alone in NB1691RV NB cells, and decreased *2DG*-induced phosphorylation and activation of Akt at T308, T450, and S473, potentiating endoplasmic reticulum (ER) stress, in NB1691, SH-SY5Y, SK-N-SH, and SK-N-BE2 cells [62]. Furthermore, Soto et al. have reported the antitumor effect of RV in combination with immunotherapy. The cotreatment with RV and hu14.18-IL2 immunocytokine (IC) showed tumor regression, necrosis areas, and a greater survival in the NB animal model, as well as RV treatment alone. In addition, RV treatment increased the levels of GD2, a disialoganglioside expressed with high density in the surface of NB cells, *in vitro*, and the infiltration of leukocytes in the tumor microenvironment in treated mice [63]. Besides RV, curcumin has been reported to be effective against NB. Curcumin is a polyphenol found in the rhizome of *Curcuma longa* (turmeric) with anti-inflammatory and antioxidative effects [64–66] and has been reported to be effective in metabolic syndrome [67], pain, osteoarthritis [68], and cancer [69]. Sidhar and Giri have reported the effective anticancer role of curcumin in NB. Indeed, curcumin treatment in N2a NB cells inhibited proliferation and induced apoptosis through the inhibition of ERK1/2 and the proteolytic activation of caspase-3, and poly (ADP-ribose) polymerase (PARP-1) cleavage and inactivation. Moreover, curcumin enhanced p53-ser15 phosphorylation and activation, which induces the expression of pro-apoptotic genes *Bex1*, *Bex2*, *Bex4*, and *Bex6* leading to the activation of the intrinsic apoptotic pathway in N2a NB cells [70]. These data proved the important role played by several nutraceutical compounds in regulating NB cancer biology.

## 6. Berberine

Berberine (BBR) is an isoquinoline quaternary alkaloid extracted from medicinal plants such as *Hydrastis canadensis*, *Coptis chinensis*, *Coptis japonica*, *Phellodendron chinense Schneid*, *Phellodendron amurense*, and *Berberis aristata* [71, 72], which displays anti-inflammatory, antioxidant, and antimicrobial effects [73, 74]. The anti-inflammatory and antioxidative effects of BBR were found to be important in the protection against type 2 diabetes, hypertension, and cardiovascular diseases [75, 76]. BBR exerts the anti-inflammatory activity by the reduction of proinflammatory cytokines and acute-phase proteins [77]. Jeong et al. have reported that BBR treatment repressed the expression of proinflammatory genes, including interleukin-6 (IL-6), interleukin-1beta (IL-1*β*), tumor necrosis factor-alpha (TNF-*α*), cyclooxygenase-2 (COX2), and matrix metalloprotease-9 (MMP-9), and decreased mitogen-activated protein kinase (MAPK) phosphorylation, while increased adenosine monophosphate-activated protein kinase (AMPK) phosphorylation and activation, leading to a decrease in pro-inflammatory responses in macrophages and adipocytes [78]. In addition, BBR is shown to block the activation of Toll-like receptor 4 (TLR4)/nuclear factor-kappa B (NF-*κ*B) and the inflammatory response in an *in vivo* model of diabetic nephropathy [79]. BBR is also a potent antioxidant agent. Indeed, BBR reduces reactive oxygen species (ROS) production, enhances the phosphorylation and activation of the endothelial nitric oxide synthase (eNOS) due to activation of AMPK, and downregulates the expression of nicotinamide adenine dinucleotide phosphate (NADPH) oxidases (NOX) 4 in a dose-dependent manner, leading to improved endothelial function in type 2 diabetes [80–82]. Moreover, BBR enhanced the expression and activity of the glucose transporter 1 (GLUT1) leading to an increased glucose consumption in an insulin-dependent manner in HepG2 hepatocellular carcinoma cell, muscle cells, and adipocytes [83–85]. Clinical and *in vivo* studies have shown that BBR administration in patients and animal model of type 2 diabetic patients, reduced plasma triglycerides, low-density lipoprotein (LDL), fasting blood glucose (FBG), postprandial blood glucose (PBG), and cholesterol [86–89]. Recent findings have suggested the efficacy of BBR in hypertension and cardiovascular disease. Guo et al. have demonstrated the effect of BBR in delaying the onset, severity, and the pathophysiology of hypertension by activating the renin-angiotensin system (RAS) and proinflammatory cytokines such as IL-6, interleukin-17 (IL-17), and interleukin-23 (IL-23) in hypertensive rats [90]. Clinical trials have reported that BBR treatment reduced the levels of the cardiovascular risk indicators, such as LDL, and apolipoprotein B/apolipoprotein A1 (ApoB/ApoA1) ratios, and improved the quality of life in congestive heart failure patients in combination with conventional therapy [91, 92].

Moreover, it has been shown that BBR could contribute to clinical benefits for neurodegenerative diseases, including Alzheimer's disease [93], Huntington's disease [94], and Parkinson's disease [95]. In Alzheimer's disease, BBR could be effective in decreasing the generation of the beta-amyloid (A*β*) peptide by APP processing in H4 human neuroglioma cells, and in inhibiting the activity of beta-site APP cleaving enzyme-1 (BACE-1) in a rabbit model of Alzheimer's disease [96, 97]. In Huntington disease, BBR reduces the accumulation of huntingtin by autophagy, alleviates motor dysfunction, and prolongs the survival of transgenic N171-82Q mice [94]. Finally, Kim et al. have showed that BBR treatment improved memory and enhanced motor balance in Parkinson's disease in a mouse model, due to a reduction in dopaminergic neurons in the substantia nigra and apoptosis in hippocampus [95]. In contrast, Kwon et al. have shown no difference in dopaminergic neuronal loss in a PC12 model of Parkinson's disease between the BBR treated group compared with the untreated [98]. All these experimental evidences confirmed the function of BBR in regulating important cell processes and suggest new therapeutic approaches in various diseases, alone, or in combination with current therapies.

## 7. Berberine and Cancer

Recent findings have demonstrated that BBR exhibits antitumor activity through the inhibition of cancer cell progression and migration and the induction of apoptosis in several types of cancer [99–102]. Abrams et al. have reported that also BBR derivatives exert anticancer proprieties; indeed the treatment with a panel of BBR derivatives (NAX compounds) in three different PDAC pancreatic cancer cell lines, inhibited proliferation, and suppressed colony formation [103]. BBR directly binds with DNA inducing double-strand breaks leading to the inhibition of gene transcription and cell cycle arrest in various human cancer cell lines [104]. Jiang et al. have reported that treatment with BBR enhanced cell cycle arrest in the G2/M phase through the increased expression of the cell cycle protein p21, in KYSE-70 human esophageal carcinoma cell line, in a dose-dependent manner [105]. In LoVo human colorectal adenocarcinoma cells, BBR dose- and time-dependent treatment downregulated cell cycle protein, such as cyclin B1, cdc25c, and cdc2, leading to cell cycle arrest in G2/M phase and suppression of colorectal adenocarcinoma cell growth [106]. Li et al. have demonstrated that the effect of BBR on the suppression of the cell cycle in the G0/G1 phase in HCC hepatocellular carcinoma cells occurred through the enhanced expression of CDKIs p21Cip1 and p27Kip1 via Akt/FocO3a/Skp2 axis regulation [107]. In U2OS, Saos-2, and Hos human osteosarcoma cells, BBR induces a cell cycle arrest in the G1 phase by a p53-dependent upregulation of p21 and cell cycle arrest in G2/M phase in a p53- independent manner [108]. Furthermore, Gao et al. have demonstrated that BBR enhanced S phase cycle arrest in MDA-MB-231 breast cancer cells, leading to a higher sensitivity of cancer cells to chemotherapy [109].

Several studies have reported the proapoptotic effect of BBR through the disruption of the mitochondrial membrane potential, leading to the inhibition of the mitochondrial respiration and mitochondrial dysfunction in various types of cancer. Meeran et al. have showed that BBR treatment-induced apoptosis through the alteration of mitochondrial membrane potential and the cleavage of caspase-3, caspase-9 protein, and PARP in PC-3 prostate cancer cells [110]. BBR significantly induces apoptosis in MDA-MB-468, HCC70, and BT-20 triple-negative breast cancer cells through the cleavage of caspase-7 and caspase-8 protein in MDA-MB-468 and BT-20 cell line, and by the cleavage of PARP in HCC70 cells [111]. In addition, Wen et al. have reported that the combination of BBR and tamoxifen (TAM) induced cell cycle arrest in the G1 phase and apoptosis through the induction of p21Cip-1 and the increase of the Bax/Bcl-2 ratio in MCF7 and tamoxifen-resistant MCF7/TAMR breast cancer cells [112]. Moreover, in HCT-116 and HT-29 colorectal cancer cells, BBR treatment induces apoptosis through the expression of nonsteroidal anti-inflammatory drug-activated *gene-1* (*NAG-1)* by PKC, ERK, and GSK-3*β* pathways, and *ATF3* in a p53-dependent manner [113]. In addition, BBR regulates cell autophagy, a programmed cell death process that plays an important role in cellular homeostasis and survival. In HepG2 and MHCC97-L hepatic carcinoma cells, BBR induces autophagic cell death by the activation of Beclin-1 (BECN-1) and the inhibition of m-TOR signaling through the downregulation of Akt activity and the upregulation of P38 MAPK signaling [114]. Wang et al. have demonstrated that cotreatment with BBR and curcumin induces accumulation of Microtubule-associated protein 1 light chain 3 beta (LC3-II) and reduction of p62, two markers for the determination of autophagy. To note, the combined treatment has been shown to be more effective than treatment with BBR or curcumin alone in enhancing the autophagy process via the JNK/Bcl-2/Beclin-1 pathway in breast cancer cells [115]. Furthermore, in U343 and MIA PaCa-2 glioblastoma cells, BBR treatment induces BECN1expression and LC3 upregulation leading to an increased autophagy [116].

**Figure 1:**
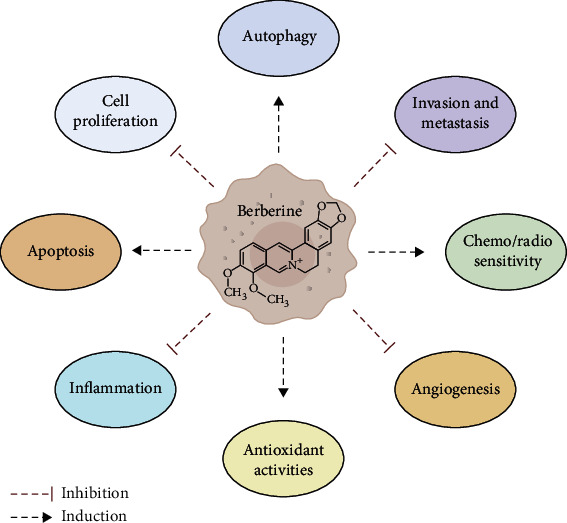
Schematic representation of cancer-related processes regulated by BBR. Figure created with BioRender.

BBR could also interfere with tumor progression, invasion, and metastatic processes in various cancer lines by modulating the expression and signaling of tumor-related protein. In triple-negative breast cancer cells, BBR suppresses cell migration by the inhibition of TGF-*β*1 expression by reduction of matrix metalloproteinase-2 (MMP-2) and MMP-9 expression [117] and by the downregulation of EGFR protein and by suppression of IL-8 expression due to the inhibition of MEK and ERK phosphorylation, leading to inhibition of the EGFR/MEK/ERK signaling and invasiveness [118]. In PC-3 and DU145 prostate cancer cells, BBR downregulates the expression of genes involved in the epithelial-mesenchymal transition (EMT), including platelet-derived growth factor receptor beta (PDGFR), bone morphogenetic protein 7 (BMP7), and collagen type I alpha 2 (COL1A2), and represses the expression of the EMT transcription factor Snail-1, with inhibition of the migratory and invasive capability of these cancer cells [119]. Moreover, Tsang et al. have reported that BBR inhibited invasiveness and angiogenesis *in vivo* and *in vitro*. In particular, BBR treatment reduced tumor growth, extra-tumor invasion, and metastatic growth of HCC xenografts. In addition, BBR downregulates HIF-1*α* and vascular endothelial growth factor (VEGF) expression and suppresses the transcription of Id-1 in HCC cells leading to inhibitions of cellular growth and invasion [120]. Taken together, all these data suggest that BBR have an important role in contrasting cancer cell growth and progression by affecting several processes (Figure 1) and reinforce the concept that this nutraceutical compound could be considered for the treatment of cancer patients, alone or in combination with standard therapies.

## 8. Berberine and NB

Treatment of NB is challenging for the clinician, due to its recurrence after chemotherapeutic therapies. Recent findings suggest that targeting NB using anticancer natural compounds is a promising strategy to improve treatment outcomes. Various studies have reported the antitumor efficacy of BBR in reducing progression and invasiveness in NB. Choi et al. have demonstrated that BBR treatment resulted in an enhanced apoptosis in SK-N-SH p53-expressing cells compared to SK-N-MC p53-deficient cells and increased the expression of the proapoptotic Bax protein, cleaved caspase-3, and cleaved PARP, while reduced the expression of the antiapoptotic Bcl-2 protein [121]. Moreover, BBR has demonstrated to be effective in EMT mechanism suppression. In Neuro2A cells, BBR suppresses EMT by the inhibition of TGF-*β* through modulation of the TGF-*β* receptors II and III, and via downregulation of the PI3K/Akt and Ras-Raf-ERK signaling and upregulation of p38 MAPK signaling. In addition, BBR inhibits EMT by the switch from the mesenchymal markers fibronectin and vimentin to the epithelial marker E-cadherin [122]. The combined treatment of BBR with curcumin results to be effective in reducing NB cell viability. The cotreatment with BBR and SLCP (solid lipid curcumin particles) increase cell death compared to BBR treatment alone in Neuro2A and SH-SY5Y NB cells, without evidence of toxicity [123]. The combination with arsenic trioxide (As_2_O_3_), as well as curcumin, and BBR has reported to improve the cytotoxic effect in NB cancer cells. Indeed, the cotreatment enhanced cell death, compared to BBR alone in a dose- and time-dependent manner, induced apoptosis through the reduction of Bcl-2, Bid, and Bcl-XL protein, and increases intracellular ROS production and lipid peroxidation in SH-SY5Y cells [124]. Recent literature data have shown the involvement of BBR in decreasing cancer stemness and promoting differentiation, representing a new possible strategy against NB progression. BBR treatment increases the expression of three neuronal differentiation markers, microtubule-associated protein 2 (MAP2), *β*-tubulin III, and neural cell adhesion molecule (NCAM), suggesting a role of BBR in neuronal differentiation in Neuro2A cells. In addition, BBR strongly downregulates the expression of the stemness marker CD133, *β*-catenin, notch2, *MYCN*, sox2, and nestin leading to a reduction of cancer stemness [122]. As previously described, BBR exhibits also neuroprotective effects against neurodegenerative diseases. Interestingly, BBR has demonstrated to be effective in the inhibition of monoamine oxidases (MAOs), one of the key enzymes implicated in the pathogenesis of Alzheimer's disease, which results widely expressed in NB [125, 126]. MAOs are a family of flavoproteins that catalyze the oxidation of amine substrates in proteins, responsible for maintaining neurotransmitter homeostasis, mitochondrial function, redox state, and are involved in cell death mechanism. Monoamine oxidase A (MAO A) and monoamine oxidase B (MAO B) are outer mitochondrial membrane proteins that oxidize amines in the corresponding imines. In particular, MAO A degrades serotonin, norepinephrine, and dopamine, while MAO B oxidizes benzylamine, dopamine, and phenylethylamine, with the production of aldehydes and hydrogen peroxide (H_2_O_2_), contributing to ROS generation [127]. In a very recent study, MAO A overexpression was found to be involved in enhancing basal ROS levels and autophagy through phosphorylation of Bcl-2, and in increasing the activity of the complex IV of the electron transport chain (ETC) without changes in ATP production in SH-SY5Y cells, suggesting a relationship between MAO A and mitochondrial function and identifying MAO A as a regulator of cell survival [126]. These experimental evidences showed the involvement of BBR and MAO A in neurodegenerative diseases and NB survival, suggesting that further investigations are needed in order to explore a new therapeutic strategy in NB disease.

## 9. Conclusions

**Figure 2:**
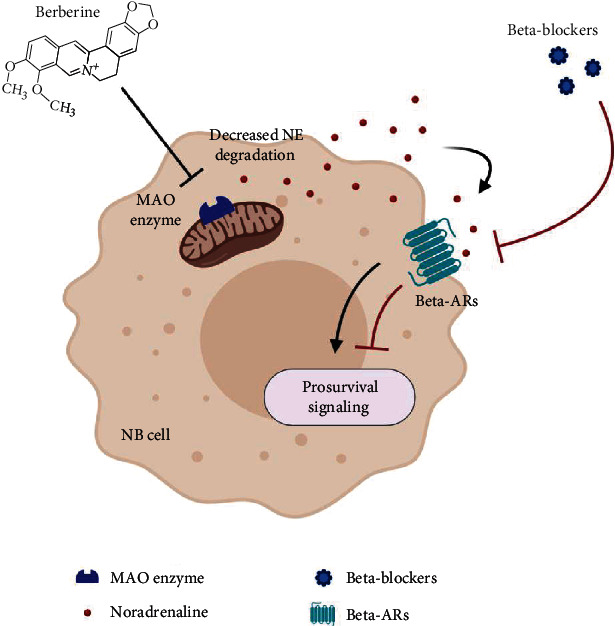
Proposed therapeutic approach to overcome side effects due to MAO A enzyme blockade by BBR consisting in concomitant administration of BBR with *β*-AR blockers. Figure created with BioRender.

This review highlights the critical role of BBR as an anticancer nutraceutical compound, regulating several signaling pathways related to cancer and, in particular, to NB progression. However, despite the countless benefits, some issues regarding the possible use of BBR in patients affected by NB have to be considered. Although BBR is effective in inhibiting some important pathways in tumor progression, its administration to cancer patients may cause few side effects. In particular, due to the MAO enzyme blockade, an increase of norepinephrine level in cells and in the tumor microenvironment could probably occur following BBR administration. The increased level of norepinephrine could therefore cause a stronger and sustained activation of *β*-ARs present in tumor NB cells leading to prosurvival signaling. Therefore, here, we propose that a promising strategy to overcome this undesired effect could be the contemporaneous assumption of BBR together with *β*-AR blockers (Figure 2).

In addition, as described above, the inhibition of MAO A contributes to the lowering of mitochondrial ROS, and the same effect has already been shown for *β*3-AR blockade in melanoma and in embryonic cells [128]. Therefore, here, we also speculate that the decrease of mitochondrial ROS production following *β*3-AR blockade relies, at least in part, on the inhibition of MAO A, and this putative synergistic effect may boost BBR beneficial action.

## References

[1] Brodeur G. M. (2003). Neuroblastoma: biological insights into a clinical enigma. *Nature Reviews Cancer*.

[2] Mueller S., Matthay K. K. (2009). Neuroblastoma: biology and staging. *Current Oncology Reports*.

[3] Trigg R. M., Turner S. D. (2018). ALK in Neuroblastoma: Biological and Therapeutic Implications. *Cancers*.

[4] Campbell K., Gastier-Foster J. M., Mann M. (2017). Association ofMYCNcopy number with clinical features, tumor biology, and outcomes in neuroblastoma: A report from the Children's Oncology Group. *Cancer*.

[5] Look A. T., Hayes F. A., Shuster J. J. (1991). Clinical relevance of tumor cell ploidy and N-myc gene amplification in childhood neuroblastoma: a pediatric oncology group study. *Journal of Clinical Oncology*.

[6] Weiss W. A., Aldape K., Mohapatra G., Feuerstein B. G., Bishop J. M. (1997). Targeted expression of MYCN causes neuroblastoma in transgenic mice. *The EMBO Journal*.

[7] Yaari S., Jacob-Hirsch J., Amariglio N., Haklai R., Rechavi G., Kloog Y. (2005). Disruption of cooperation between Ras and MycN in human neuroblastoma cells promotes growth arrest. *Clinical Cancer Research*.

[8] Tonelli R., Purgato S., Camerin C. (2005). Anti-gene peptide nucleic acid specifically inhibits MYCN expression in human neuroblastoma cells leading to cell growth inhibition and apoptosis. *Molecular Cancer Therapeutics*.

[9] Chipumuro E., Marco E., Christensen C. L. (2014). CDK7 Inhibition Suppresses Super-Enhancer-Linked Oncogenic Transcription in MYCN-Driven Cancer. *Cell*.

[10] Garaventa A., Parodi S., de Bernardi B. (2009). Outcome of children with neuroblastoma after progression or relapse. A retrospective study of the Italian neuroblastoma registry. *European Journal of Cancer*.

[11] Kushner B. H., Modak S., Kramer K. (2014). Striking dichotomy in outcome of MYCN-amplified neuroblastoma in the contemporary era. *Cancer*.

[12] Schleiermacher G., Javanmardi N., Bernard V. (2014). Emergence of new ALK mutations at relapse of neuroblastoma. *Journal of Clinical Oncology*.

[13] Padovan-Merhar O. M., Raman P., Ostrovnaya I. (2016). Enrichment of targetable mutations in the relapsed neuroblastoma genome. *PLoS Genetics*.

[14] Zhu S., Lee J. S., Guo F. (2012). Activated ALK collaborates with MYCN in neuroblastoma pathogenesis. *Cancer Cell*.

[15] Berry T., Luther W., Bhatnagar N. (2012). The _ALK^F1174L^_ Mutation Potentiates the Oncogenic Activity of MYCN in Neuroblastoma. *Cancer Cell*.

[16] Schönherr C., Ruuth K., Kamaraj S. (2012). Anaplastic Lymphoma Kinase (ALK) regulates initiation of transcription of _MYCN_ in neuroblastoma cells. *Oncogene*.

[17] De Brouwer S., De Preter K., Kumps C. (2010). Meta-analysis of neuroblastomas reveals a skewed ALK mutation spectrum in tumors with MYCN amplification. *Clinical Cancer Research*.

[18] Monclair T., Brodeur G. M., Ambros P. F. (2009). The international neuroblastoma risk group (INRG) staging system: an INRG task force report. *Journal of Clinical Oncology*.

[19] Lands A. M., Arnold A., Mcauliff J. P., Luduena F. P., Brown T. G. (1967). Differentiation of receptor systems activated by sympathomimetic amines. *Nature*.

[20] Emorine L., Marullo S., Briend-Sutren M. (1989). Molecular characterization of the human beta 3-adrenergic receptor. *Science*.

[21] Michel M. C., Gravas S. (2016). Safety and tolerability of *β*3-adrenoceptor agonists in the treatment of overactive bladder syndrome - insight from transcriptosome and experimental studies. *Expert Opinion on Drug Safety*.

[22] Cernecka H., Sand C., Michel M. C. (2014). The odd sibling: features of*β*3-Adrenoceptor pharmacology. *Molecular Pharmacology*.

[23] Michel M. C., Bond R. A., Summers R. J. (2019). Adrenoceptors—new roles for old players. *British Journal of Pharmacology*.

[24] Schuller H. M., Cole B. (1989). Regulation of cell proliferation by *β*-adrenergjc receptors in a human lung adenocarcinoma cell line. *Carcinogenesis*.

[25] Antoni M. H., Lutgendorf S. K., Cole S. W. (2006). The influence of bio-behavioural factors on tumour biology: pathways and mechanisms. *Nature Reviews. Cancer*.

[26] Cole S. W., Sood A. K. (2012). Molecular pathways: beta-adrenergic signaling in cancer: figure 1. *Clinical Cancer Research*.

[27] Léauté-Labrèze C., de la Roque E. D., Hubiche T., Boralevi F., Thambo J. B., Taïeb A. (2009). Propranolol for severe hemangiomas of infancy. *The New England Journal of Medicine*.

[28] Stiles J., Amaya C., Pham R. (2012). Propranolol treatment of infantile hemangioma endothelial cells: a molecular analysis. *Experimental and Therapeutic Medicine*.

[29] Montoya A., Amaya C. N., Belmont A. (2017). Use of non-selective *β*-blockers is associated with decreased tumor proliferative indices in early stage breast cancer. *Oncotarget*.

[30] Sood A. K., Bhatty R., Kamat A. A. (2006). Stress hormone-mediated invasion of ovarian cancer cells. *Clinical Cancer Research*.

[31] Watkins J. L., Thaker P. H., Nick A. M. (2015). Clinical impact of selective and nonselective beta-blockers on survival in patients with ovarian cancer. *Cancer*.

[32] Moretti S., Massi D., Farini V. (2013). _*β*_ -adrenoceptors are upregulated in human melanoma and their activation releases pro-tumorigenic cytokines and metalloproteases in melanoma cell lines. *Laboratory Investigation*.

[33] Calvani M., Pelon F., Comito G. (2015). Norepinephrine promotes tumor microenvironment reactivity through *β*3-adrenoreceptors during melanoma progression. *Oncotarget*.

[34] Perrone M. G., Notarnicola M., Caruso M. G., Tutino V., Scilimati A. (2008). Upregulation of *β*_3_-Adrenergic receptor mRNA in human colon cancer: a preliminary study. *Oncology*.

[35] Goldfarb Y., Sorski L., Benish M., Levi B., Melamed R., Ben-Eliyahu S. (2011). Improving postoperative immune status and resistance to cancer Metastasiss. *Annals of Surgery*.

[36] Sloan E. K., Priceman S. J., Cox B. F. (2010). The sympathetic nervous system induces a metastatic switch in primary breast cancer. *Cancer Research*.

[37] Palm D., Lang K., Niggemann B. (2006). The norepinephrine-driven metastasis development of PC-3 human prostate cancer cells in BALB/c nude mice is inhibited by *β*-blockers. *International Journal of Cancer*.

[38] Inbar S., Neeman E., Avraham R., Benish M., Rosenne E., Ben-Eliyahu S. (2011). Do stress responses promote leukemia progression? An animal study suggesting a role for epinephrine and prostaglandin-E2 through reduced NK activity. *PLoS One*.

[39] Thaker P. H., Han L. Y., Kamat A. A. (2006). Chronic stress promotes tumor growth and angiogenesis in a mouse model of ovarian carcinoma. *Nature Medicine*.

[40] Hassan S., Karpova Y., Flores A., D’Agostino R., Kulik G. (2013). Surgical stress delays prostate involution in mice. *PLoS One*.

[41] Zahalka A. H., Arnal-Estapé A., Maryanovich M. (2017). Adrenergic nerves activate an angio-metabolic switch in prostate cancer. *Science*.

[42] Pérez-Sayáns M., Somoza-Martín J. M., Barros-Angueira F., Diz P. G., Gándara Rey J. M., García-García A. (2010). *β*-Adrenergic receptors in cancer: therapeutic implications. *Oncology Resarch*.

[43] Huang X. E., Hamajima N., Saito T. (2001). Possible association of beta2- and beta3-adrenergic receptor gene polymorphisms with susceptibility to breast cancer. *Breast Cancer Research*.

[44] Babol K., Przybylowska K., Lukaszek M., Pertynski T., Blasiak J. (2004). An association between the Trp64Arg polymorphism in the beta3-adrenergic receptor gene and endometrial cancer and obesity. *Journal of Experimental & Clinical Cancer Research*.

[45] Rai R., Kim J., Misra S., Kumar A., Mittal B. (2015). A multiple interaction analysis reveals ADRB3 as a potential candidate for gallbladder cancer predisposition via a complex interaction with other candidate gene variations. *International Journal of Molecular Sciences*.

[46] Magnon C., Hall S. J., Lin J. (2013). Autonomic nerve development contributes to prostate cancer progression. *Science*.

[47] Dal Monte M., Casini G., Filippi L., Nicchia G. P., Svelto M., Bagnoli P. (2013). Functional involvement of *β*3-adrenergic receptors in melanoma growth and vascularization. *Journal of Molecular Medicine*.

[48] Calvani M., Bruno G., Dal Monte M. (2019). *β*3‐Adrenoceptor as a potential immuno-suppressor agent in melanoma. *British Journal of Pharmacology*.

[49] Brodeur G. M., Pritchard J., Berthold F. (1993). Revisions of the international criteria for neuroblastoma diagnosis, staging, and response to treatment. *Journal of Clinical Oncology*.

[50] Pritchard J., Barnes J., Germond S. (1989). Stage and urinary catecholamine metabolite excretion in neuroblastoma. *Lancet*.

[51] Wolter J. K., Wolter N. E., Blanch A. (2014). Anti-tumor activity of the beta-adrenergic receptor antagonist propranolol in neuroblastoma. *Oncotarget*.

[52] Pasquier E., Street J., Pouchy C. (2013). _*β*_ -blockers increase response to chemotherapy via direct antitumour and anti-angiogenic mechanisms in neuroblastoma. *British Journal of Cancer*.

[53] Bruno G., Cencetti F., Pini A. (2020). *Β*3-adrenoreceptor blockade reduces tumor growth and increases neuronal differentiation in neuroblastoma via SK2/S1P_2_ modulation. *Oncogene*.

[54] Laverdière C., Liu Q., Yasui Y. (2009). Long-term outcomes in survivors of neuroblastoma: a report from the childhood cancer survivor study. *JNCI Journal of the National Cancer Institute*.

[55] Su D., Cheng Y., Liu M. (2013). Comparision of piceid and resveratrol in antioxidation and antiproliferation activities in vitro. *PLoS ONE*.

[56] Wu J. M., Hsieh T. C., Wang Z. (2011). Cardioprotection by resveratrol: a review of effects/targets in cultured cells and animal tissues. *American Journal of Cardiovascular Disease*.

[57] Park S., Lim J., Kim J. R., Cho S. (2017). Inhibitory effects of resveratrol on hepatitis B virus X protein-induced hepatocellular carcinoma. *Journal of Veterinary Science*.

[58] Rimando A., Suh N. (2008). Biological/chemopreventive activity of stilbenes and their effect on colon cancer. *Planta Medica*.

[59] Fuggetta M. P., D??Atri S., Lanzilli G. (2004). In vitro antitumour activity of resveratrol in human melanoma cells sensitive or resistant to temozolomide. *Melanoma Research*.

[60] van Ginkel P. R., Sareen D., Subramanian L. (2007). Resveratrol inhibits tumor growth of human neuroblastoma and mediates apoptosis by directly targeting mitochondria. *Clinical Cancer Research*.

[61] Rahman M. A., Kim N. H., Kim S. H., Oh S. M., Huh S. O. (2012). Antiproliferative and cytotoxic effects of resveratrol in mitochondria-mediated apoptosis in rat B103 neuroblastoma cells. *The Korean Journal of Physiology & Pharmacology*.

[62] Graham R. M., Hernandez F., Puerta N., de Angulo G., Webster K. A., Vanni S. (2016). Resveratrol augments ER stress and the cytotoxic effects of glycolytic inhibition in neuroblastoma by downregulating Akt in a mechanism independent of SIRT1. *Experimental & Molecular Medicine*.

[63] Soto B. L., Hank J. A., Van De Voort T. J. (2011). The anti-tumor effect of resveratrol alone or in combination with immunotherapy in a neuroblastoma model. *Cancer Immunology, Immunotherapy*.

[64] Kuptniratsaikul V., Dajpratham P., Taechaarpornkul W. (2014). Efficacy and safety of Curcuma domestica extracts compared with ibuprofen in patients with knee osteoarthritis: a multicenter study. *Clinical Intervention in Aging*.

[65] Aggarwal B. B., Harikumar K. B. (2009). Potential therapeutic effects of curcumin, the anti-inflammatory agent, against neurodegenerative, cardiovascular, pulmonary, metabolic, autoimmune and neoplastic diseases. *The International Journal of Biochemistry & Cell Biology*.

[66] Sahebkar A., Serban M.-C., Ursoniu S., Banach M. (2015). Effect of curcuminoids on oxidative stress: A systematic review and meta- analysis of randomized controlled trials. *Journal of Functional Foods*.

[67] Panahi Y., Hosseini M. S., Khalili N. (2016). Effects of curcumin on serum cytokine concentrations in subjects with metabolic syndrome: a post-hoc analysis of a randomized controlled trial. *Biomedicine & Pharmacotherapy*.

[68] Henrotin Y., Priem F., Mobasheri A. (2013). Curcumin: a new paradigm and therapeutic opportunity for the treatment of osteoarthritis: curcumin for osteoarthritis management. *Springer Plus*.

[69] Wright L., Frye J., Gorti B., Timmermann B., Funk J. (2013). Bioactivity of turmeric-derived curcuminoids and related metabolites in breast cancer. *Current Pharmaceutical Design*.

[70] Sidhar H., Giri R. K. (2017). Induction of _Bex_ genes by curcumin is associated with apoptosis and activation of p53 in N2a neuroblastoma cells. *Scientific Reports*.

[71] Chen X.-W., Di Y. M., Zhang J., Zhou Z.-W., Li C. G., Zhou S.-F. (2012). Interaction of herbal compounds with biological targets: a case study with berberine. *The Scientific World Journal*.

[72] Tillhon M., Guamán Ortiz L. M., Lombardi P., Scovassi A. I. (2012). Berberine: new perspectives for old remedies. *Biochemical Pharmacology*.

[73] Duan Y., Liu T., Zhou Y., Dou T., Yang Q. (2018). *Glycoside hydrolase family 18 and 20 enzymes are novel targets of the traditional medicine berberine*. *Journal of Biological Chemistry*.

[74] McCubrey J. A., Lertpiriyapong K., Steelman L. S. (2017). Effects of resveratrol, curcumin, berberine and other nutraceuticals on aging, cancer development, cancer stem cells and microRNAs. *Aging (Albany NY)*.

[75] Yin J., Xing H., Ye J. (2008). Efficacy of berberine in patients with type 2 diabetes mellitus. *Metabolism*.

[76] Affuso F., Mercurio V., Fazio V., Fazio S. (2010). Cardiovascular and metabolic effects of berberine. *World Journal of Cardiology*.

[77] Xie X., Chang X., Chen L. (2013). Berberine ameliorates experimental diabetes-induced renal inflammation and fibronectin by inhibiting the activation of RhoA/ROCK signaling. *Molecular and Cellular Endocrinology*.

[78] Jeong H. W., Hsu K. C., Lee J. W. (2009). Berberine suppresses proinflammatory responses through AMPK activation in macrophages. *American Journal of Physiology. Endocrinology and Metabolism*.

[79] Zhu L., Han J., Yuan R., Xue L., Pang W. (2018). Berberine ameliorates diabetic nephropathy by inhibiting TLR4/NF-*κ*B pathway. *Biological Research*.

[80] Wang C., Li J., Lv X. (2009). Ameliorative effect of berberine on endothelial dysfunction in diabetic rats induced by high-fat diet and streptozotocin. *European Journal of Pharmacology*.

[81] Wang Y., Huang Y., Lam K. S. L. (2009). Berberine prevents hyperglycemia-induced endothelial injury and enhances vasodilatation via adenosine monophosphate-activated protein kinase and endothelial nitric oxide synthase. *Cardiovascular Research*.

[82] Zhang L. S., Zhang J. H., Feng R. (2019). Efficacy and safety of berberine alone or combined with statins for the treatment of hyperlipidemia: a systematic review and meta-analysis of randomized controlled clinical trials. *The American Journal of Chinise Medicine*.

[83] Kim S. H., Shin E. J., Kim E. D., Bayaraa T., Frost S. C., Hyun C. K. (2007). Berberine activates GLUT1-mediated glucose uptake in 3T3-L1 adipocytes. *Biological and Pharmaceutical Bulletin*.

[84] Zhou L., Yang Y., Wang X. (2007). Berberine stimulates glucose transport through a mechanism distinct from insulin. *Metabolism*.

[85] Cok A., Plaisier C., Salie M. J., Oram D. S., Chenge J., Louters L. L. (2011). Berberine acutely activates the glucose transport activity of GLUT1. *Biochimie*.

[86] Kong W., Wei J., Abidi P. (2004). Berberine is a novel cholesterol-lowering drug working through a unique mechanism distinct from statins. *Nature Medicine*.

[87] Lee Y. S., Kim W. S., Kim K. H. (2006). Berberine, a natural plant product, activates AMP-activated protein kinase with beneficial metabolic effects in diabetic and insulin-resistant states. *Diabetes*.

[88] Leng S. H., Lu F. E., Xu J. J. (2004). Therapeutic effects of berberine in impaired glucose tolerance rats and its influence on insulin secretion. *Acta Pharmacologica Sinca*.

[89] Tang L. Q., Wei W., Chen L. M., Liu S. (2006). Effects of berberine on diabetes induced by alloxan and a high-fat/high- cholesterol diet in rats. *Journal of Ethnopharmacology*.

[90] Zuo F., Nakamura N., Akao T., Hattori M. (2006). Pharmacokinetics of berberine and its main metabolites in conventional and pseudo germ-free rats determined by liquid chromatography/ion trap mass spectrometry. *Drug Metabolism and Disposition: The Biological Fate of Chemicals*.

[91] Solà R., Valls R.-M., Puzo J. (2014). Effects of poly-bioactive compounds on lipid profile and body weight in a moderately hypercholesterolemic population with low cardiovascular disease risk: a multicenter randomized Trial. *PLoS One*.

[92] Zeng X. H., Zeng X. J., Li Y. Y. (2003). Efficacy and safety of _berberine_ for congestive heart failure secondary to ischemic or idiopathic dilated cardiomyopathy. *The American Journal of Cardiology*.

[93] Ahmed T., Gilani A.-u.-H., Abdollahi M., Daglia M., Nabavi S. F., Nabavi S. M. (2015). Berberine and neurodegeneration: a review of literature. *Pharmacological Reports*.

[94] Jiang W., Wei W., Gaertig M. A., Li S., Li X. J. (2015). Therapeutic effect of berberine on Huntington’s disease transgenic mouse model. *PLoS One*.

[95] Kim M., Cho K.-H., Shin M.-S. (2014). Berberine prevents nigrostriatal dopaminergic neuronal loss and suppresses hippocampal apoptosis in mice with Parkinson’s disease. *International Journal of Molecular Medicine*.

[96] Panahi N., Mahmoudian M., Mortazavi P., Hashjin G. S. (2013). Experimental research Effects of berberine on *β*-secretase activity in a rabbit model of Alzheimer’s disease. *Archives of Medical Sciencie: AMS*.

[97] Asai M., Iwata N., Yoshikawa A. (2007). Berberine alters the processing of Alzheimer's amyloid precursor protein to decrease A*β* secretion. *Biochemical and Biophysical Research Communication*.

[98] Kwon I. H., Choi H. S., Shin K. S. (2010). Effects of berberine on 6-hydroxydopamine-induced neurotoxicity in PC12 cells and a rat model of Parkinson's disease. *Neuroscience Letters*.

[99] Wang Y., Zhang S. (2018). Berberine suppresses growth and metastasis of endometrial cancer cells via miR-101/COX-2. *Biomedicine & Pharmacotherapy*.

[100] Ahmadiankia N., Moghaddam H. K., Mishan M. A. (2016). Berberine suppresses migration of MCF-7 breast cancer cells through down-regulation of chemokine receptors. *Iranian Journal of Basic Medical Science*.

[101] Xie J., Xu Y., Huang X. (2015). Berberine-induced apoptosis in human breast cancer cells is mediated by reactive oxygen species generation and mitochondrial-related apoptotic pathway. *Tumour Biology: the journal of the International Society for Oncodevelopmental Biology and Medicine*.

[102] Chen Q. Q., Shi J. M., Ding Z. (2019). Berberine induces apoptosis in non-small-cell lung cancer cells by upregulating miR-19a targeting tissue factor. *Cancer Management and Research*.

[103] Abrams S. L., Follo M. Y., Steelman L. S. (2019). Abilities of berberine and chemically modified berberines to inhibit proliferation of pancreatic cancer cells. *Advances in Biological regulation*.

[104] Wang Y., Kheir M. M., Chai Y. (2011). Comprehensive study in the inhibitory effect of berberine on gene transcription, including TATA box. *PLoS One*.

[105] Jiang S. X., Qi B., Yao W. J. (2017). Berberine displays antitumor activity in esophageal cancer *cellsin vitro*. *World Journal of Gastroenterology*.

[106] Cai Y., Xia Q., Luo R. (2014). Berberine inhibits the growth of human colorectal adenocarcinoma in vitro and in vivo. *Journal of Natural Medicines*.

[107] Li F., Dong X., Lin P., Jiang J. (2018). Regulation of Akt/FoxO3a/Skp2 Axis Is Critically Involved in Berberine-Induced Cell Cycle Arrest in Hepatocellular Carcinoma Cells. *International Journal of Molecular Science*.

[108] Liu Z., Liu Q., Xu B. (2009). Berberine induces p53-dependent cell cycle arrest and apoptosis of human osteosarcoma cells by inflicting DNA damage. *Mutation Research*.

[109] Gao X., Wang J., Li M. (2019). Berberine attenuates XRCC1-mediated base excision repair and sensitizes breast cancer cells to the chemotherapeutic drugs. *Journal of Cellular and Molecular Medicine*.

[110] Meeran S. M., Katiyar S., Katiyar S. K. (2008). Berberine-induced apoptosis in human prostate cancer cells is initiated by reactive oxygen species generation. *Toxicology and Applied Pharmacology*.

[111] El Khalki L., Maire V., Dubois T., Zyad A. (2020). Berberine Impairs the Survival of Triple Negative Breast Cancer Cells: Cellular and Molecular Analyses. *Molecules*.

[112] Wen C., Wu L., Fu L., Zhang X., Zhou H. (2016). Berberine enhances the anti-tumor activity of tamoxifen in drug-sensitive MCF-7 and drug-resistant MCF-7/TAM cells. *Molecular Medicine Reports*.

[113] Piyanuch R., Sukhthankar M., Wandee G., Baek S. J. (2007). Berberine, a natural isoquinoline alkaloid, induces NAG-1 and ATF3 expression in human colorectal cancer cells. *Cancer Letters*.

[114] Wang N., Feng Y., Zhu M. (2010). Berberine induces autophagic cell death and mitochondrial apoptosis in liver cancer cells: the cellular mechanism. *Journal of Cellular Biochemistry*.

[115] Wang K., Zhang C., Bao J. (2016). Synergistic chemopreventive effects of curcumin and berberine on human breast cancer cells through induction of apoptosis and autophagic cell death. *Scientific Reports*.

[116] Agnarelli A., Natali M., Garcia-Gil M. (2018). Cell-specific pattern of berberine pleiotropic effects on different human cell lines. *Scientific Reports*.

[117] Kim S., Lee J., You D. (2018). Berberine suppresses cell motility through downregulation of TGF-*β*1 in triple negative breast cancer cells. *Cellular Physiology and Biochemistry*.

[118] Kim S., You D., Jeong Y. (2018). Berberine down-regulates IL-8 expression through inhibition of the EGFR/MEK/ERK pathway in triple-negative breast cancer cells. *Phytomedicine*.

[119] Liu C. H., Tang W. C., Sia P. (2015). Berberine inhibits the metastatic ability of prostate cancer cells by suppressing epithelial-to-mesenchymal transition (EMT)-associated genes with predictive and prognostic relevance. *International Journal of Medical Sciences*.

[120] Tsang C. M., Cheung K. C. P., Cheung Y. C. (2015). Berberine suppresses Id-1 expression and inhibits the growth and development of lung metastases in hepatocellular carcinoma. *Biochimica et Biophysica Acta*.

[121] Choi M. S., Yuk D. Y., Oh J. H. (2008). Berberine inhibits human neuroblastoma cell growth through induction of p53-dependent apoptosis. *Anticancer Research*.

[122] Naveen C. R., Gaikwad S., Agrawal-Rajput R. (2016). Berberine induces neuronal differentiation through inhibition of cancer stemness and epithelial-mesenchymal transition in neuroblastoma cells. *Phytomedicine*.

[123] Maiti P., Plemmons A., Dunbar G. L. (2019). Combination treatment of berberine and solid lipid curcumin particles increased cell death and inhibited PI3K/Akt/mTOR pathway of human cultured glioblastoma cells more effectively than did individual treatments. *PLoS One*.

[124] Kim D. W., Ahan S. H., Kim T. Y. (2007). Enhancement of arsenic trioxide (As_2_O_3_)- mediated apoptosis using berberine in human neuroblastoma SH-SY5Y cells. *Journal of Korean Neurosurgical Society*.

[125] Ji H.-F., Shen L. (2012). Molecular basis of inhibitory activities of berberine against pathogenic enzymes in Alzheimer's disease. *The Scientific World Journal*.

[126] Ugun-Klusek A., Theodosi T. S., Fitzgerald J. C. (2019). Monoamine oxidase-A promotes protective autophagy in human SH-SY5Y neuroblastoma cells through Bcl-2 phosphorylation. *Redox Biology*.

[127] Fowler C. J., Benedetti M. S. (1983). The metabolism of dopamine by both forms of monoamine oxidase in the rat brain and its inhibition by cimoxatone. *Journal of Neurochemistry*.

[128] Calvani M., Cavallini L., Tondo A. (2018). *Β*3-adrenoreceptors control mitochondrial dormancy in melanoma and embryonic stem cells. *Oxidative Medicine and Cellular Longevity*.

